# The MOWER (middle of the week everyone gets a re-chart) pilot study: reducing in-hospital charting error with a multi-intervention

**DOI:** 10.1186/s12913-019-4230-y

**Published:** 2019-06-20

**Authors:** Tony Floyd, Siri Mårtensson, Jannine Bailey, Derek Kay, Bruce McGarity, Bronwyn K. Brew

**Affiliations:** 1 NSW Department of Health, Sydney, NSW Australia; 2 Department of Medical Epidemiology and Biostatistics, Karolinska Institutet, Stockholm, Sweden; 3 Bathurst Rural Clinical School, Western Sydney University, PO Box 9008, Bathurst, NSW 2795 Australia

**Keywords:** Inpatients, Prescriptions, Medication charts, Medical errors, Intervention

## Abstract

**Background:**

Medication charting errors occur often and can be harmful for patients. Interventions to improve charting errors have demonstrated some success particularly if the intervention uses multiple approaches including an education component. The aim of this pilot study was to determine whether a multi-faceted intervention, including education of junior doctors and weekday re-charting could reduce in-hospital charting error.

**Methods:**

Medication charts (*n* = 579) of all patients admitted to the medical ward of a medium sized regionally-based hospital in Australia over nine months (baseline and during intervention) were inspected for errors. The intervention ran for three months and involved implementation of a National Inpatient Medication Chart targeted error tool with eight targeted charting requirements which was used for visual reminders in the ward and training of junior doctors. In addition, mid-weekly re-charting (MOWER) was performed by a senior and junior doctor team.

**Results:**

The mean number of charting requirement errors significantly reduced during the intervention by 26% from 4.6 ± 1.3 to 3.4 ± 1.7 per chart (*p* < 0.001). Re-chart errors reduced on average by 50% (4.4 ± 1.4 to 2.2 ± 1.7 per chart, p < 0.001) and primary (initial) charts by 20% (4.6 ± 1.3 to 3.7 ± 1.5 per chart, p < 0.001) during the intervention. Failing to provide indication information for a drug, prescriber name, and failing to use generic rather than brand names were the categories with the most errors at baseline and also showed the largest error reductions during the intervention.

**Conclusions:**

A multi-intervention including education of junior doctors, visual reminders and midweek re-charting are effective in reducing the rate of charting errors. We advise that a larger study is now conducted using the same multi-intervention strategy in different ward settings to evaluate feasibility and sustainability of this intervention.

**Electronic supplementary material:**

The online version of this article (10.1186/s12913-019-4230-y) contains supplementary material, which is available to authorized users.

## Background

Medication charting error can result in prescribing errors which are potentially harmful, can lengthen hospital stay, and increase the financial burden to families and society [1, 2]. Prescription errors are estimated to affect 50% of hospital admissions [3], and the majority are most likely not reported [4]. The fast-growing numbers of multi-morbid, elderly patients are most at risk, as they are often prone to polypharmacy and have a higher incidence of dementia, therefore may be less aware if they are receiving the wrong medication [5, 6].

The causes behind charting and prescribing errors are multifactorial but have similar aspects from setting to setting, including heavy work load, lack of proper communication, and a hospital culture that views prescribing as a low-risk, unimportant chore [1, 7, 8]. In addition, junior doctors are often given the task of writing the medication charts despite being more likely to make prescribing errors due to insufficient knowledge and training in prescribing [1, 9]. Junior doctors report insecurity in how to prescribe [10], reluctance to oppose senior colleagues regarding medication decisions [1] and a need for more extensive pharmacology training in medical school [11, 12].

Interventions with multiple approaches such as chart training and physical reminders have been found more successful than those focusing on a single factor [13, 14]. Single factor interventions that have shown success when combined with education interventions include: audit and feedback [14], manual reminders such as posters with checklists for correct prescribing [14, 15] and involving pharmacists in medication charting and medical reconciliation [16].

In the current study, we implemented a multi-intervention strategy incorporating previously proven methods such as training of junior doctors [17], manual visual reminders [14], and a novel method involving weekday re-charting by the junior doctor under supervision of the registrar - the ‘MOWER’ (Middle Of the Week Everyone Gets a Re-chart) intervention. The aim of the multi-intervention was to raise awareness of the importance of charting practices and medication errors. The specific goal of the MOWER was to improve re-charting which can be prone to transcription error [18] by scheduling it for a weekday, therefore ensuring the primary team did the re-charting whilst engaging more experienced doctors to guide and educate the junior doctors. The idea of re-charting is consistent with proven audit and feedback interventions [14], as the medication chart is being checked and the junior doctor is receiving immediate feedback and instruction.

The main objective of our study was to determine whether a multifactorial intervention focusing on weekday team re-charting reduced charting error.

## Methods

### Study design

This study is a pilot study performed in the Medical Ward of a medium-sized hospital in regional Australia between May 2015 and January 2016 (inclusive). The general medical ward has 20 beds and there are no separate sub-specialty medical wards in the hospital. Patients are predominantly elderly patients with multiple co-morbidities and commonly present with respiratory distress. The study subjects are junior doctors (post graduate years 1 and 2) and their supervising doctors who are typically in post graduate years 3 and 4.

The focus of this study is the National Inpatient Medication Chart (NIMC) that is now implemented by the Australian Commission on Safety and Quality in Health Care for mandatory use in all hospitals in Australia, for both paper and electronic charting [19]. The NIMC is a standardized tool for communicating patient medication information consistently between health professionals and settings. The paper chart is a single page double-sided card documenting medication orders as well as medication history and adverse drug reactions. Although the NIMC was introduced to reduce charting errors and to minimize the risk of adverse medical events [20] there is disagreement on whether it has achieved these goals [18]. Paper charting continues to be used in regional Australian hospitals as well as other parts of the world [21].

### Multi-intervention

The multi-intervention ran for three months (November 2015 to January 2016) and included: 1) the display of an NIMC targeted error tool, 2) training of junior doctors and 3) a change in re-charting routine to always occur on a weekday by a team of junior and senior doctors.

#### The NIMC targeted error tool

The NIMC Targeted Error Tool (Additional file 1) was developed by the authors based on experience, scientific literature [22, 23], and an audit of ten randomly selected charts from the Medical Ward. This tool provided the operational criteria for charting errors used in this study. The error tool targeted eight specific NIMC charting requirements that met at least two of the following criteria: 1) a common cause of error, 2) potentially could cause significant harm, 3) concerned accountability, 4) did not have multiple possible entries. A description of each of the correct charting requirements is listed in Table 1. A ‘charting requirement error’ is defined as a hand-written error in any one of these charting requirements as identified on the medication chart. The error tool was introduced to staff week 1 of the intervention and displayed as a laminated poster in prominent areas throughout the ward as a visual reminder for the intervention period.

**Table 1:** Descriptions for the eight targeted charting requirements

Charting Requirement	Further description
Chart Number	The number of the NIMC in the sequence of active NIMCs is written on the front of the chart
Patient Surname	Patient’s surname handwritten below the patient label
Prescriber Name	A prescriber name was printed and legible (on at least one of the records on the page) for regular medication orders
Dated and Signed	All regular medication orders were dated and signed
Generic REG	All regular (REG) medication orders used a generic medication name rather than a brand name. Brand name exceptions: Movicol, Oxycontin, Endone, Fleet Enema
Indication REG	An indication was recorded for some regular (REG) medications ie antibiotics, anticoagulants and steroids including eye drops and topical creams.
Indication PRN	An indication was recorded for all Pro Re Nata *(*PRN, as needed) medications
Max Dosage PRN	The maximum dose in 24 h was indicated for all PRN medications

#### Training of junior doctors

A 30-min, one-on-one training session using the NIMC Targeted Error tool as a check list was provided by the senior doctor for each of four junior doctors already present on the Medical Ward (week 1 of the intervention), and for a further four junior doctors who began during the term of the intervention (week 5 of the intervention). The same senior doctor provided all training.

#### Weekday team re-charting - MOWER

In the baseline (pre-intervention) period, re-charting of NIMC charts routinely occurred seven days after admission (when space on the primary chart runs out), irrespective of the day and whether it was on the weekend or weekday. There was no structured process for completing re-charting. The intervention involved a structured approach with a junior and senior doctor team re-charting every chart, regardless of stay length, on a chosen weekday (every Wednesday). This was to prevent weekend re-charting when fewer and less experienced staff may be present, and to set up a routine for weekly team re-charting, giving priority to charting and an opportunity for further training of junior doctors. An exception was made if the patient was admitted on a Monday or Tuesday and was expected to be short stay, in these cases the team would review the primary chart, and if necessary make corrections. Team re-charting started week 1 of the intervention and continued every week for three months. The same senior doctor who did the training supervised the re-charting and was assisted by the chief pharmacist in the first few weeks.

### Data collection

Data collection occurred by reviewing hospital medication charts retrospectively and assessing them for errors according to eight charting requirements as described in Table 1 and in the section *The NIMC Targeted Error Tool*. Medication charts were included in the study if the patient had been admitted by a general physician in the Medical Ward and if the stay length was one night or more. A total of 800 medication charts were assessed for eligibility, out of which 579 were considered to be eligible and were included for further analysis. This included 420 baseline charts over six months and 159 charts during the intervention period over three months. If a charting requirement was not met, even just once, then this was recorded as a fail for that requirement.

The primary outcome measure was the average error rate per medication chart in the pre-intervention period versus the intervention period. These were then stratified into weekday and weekend charts, and primary charts versus re-charts. Secondary outcomes were the proportion of charts with an error for each of the eight charting requirements..

### Statistical analysis

For the primary outcome of charting error rates, independent samples t-tests were used to compare pre-intervention and intervention error rates for normally distributed data, otherwise Mann-Whitney U-tests were used. Pearson’s chi-square test (Fisher’s exact test where appropriate) was performed to compare the proportion of charts containing errors for the eight charting requirements (secondary outcomes). A *p*-value of < 0.05 was considered significant. SPSS version 22 (IBM Inc., New York, USA) was used for all analyses.

Ethical approval was granted from the Greater Western Human Research Ethics Committee (LNR/15/GWAHS70), with reciprocal approval granted by the Western Sydney University Human Research Ethics Committee (H11399).

## Results

Of the 420 charts audited at baseline, 23% (*n* = 97) were taken on the weekend and 77% (*n* = 323) during the week. Due to re-charting on weekdays, charting on the weekend reduced to 17% during the intervention (*n* = 27). The proportion of re-charts was constant throughout the study at 20% of all charts. However, as expected due to mandatory re-charting on a weekday, the number of re-charts that naturally fell on a weekend decreased from 28% (*n* = 24) of all re-charts to 6% (n = 2) during the intervention.

At baseline, there was an average charting requirement error rate of 4.6 ± SD 1.3 per chart, this equates to a 58% error rate per chart for the 8 charting requirements (Table 2). The multi-intervention significantly reduced the overall error rate by 26%, 1.2 (95%CI 0.9, 1.5) errors per chart during the intervention. Similarly, the weekday chart errors during the intervention reduced by 27% per chart (1.2 (95%CI 0.9, 1.5) errors, *p* < 0.001; Table 2). Weekend chart error did not significantly reduce. The re-charts made the most improvement of any sub-group during the intervention with a 50% reduction from 4.4 ± 1.4 charting requirement errors to 2.2 ± 1.7 per chart. This was more than double the reduction seen in the primary charts, which showed a 20% reduction, or 0.9 (95%CI 0.6, 1.2) errors per chart (Table 2).

**Table 2:** Charting requirement error rate per type of chart, baseline and during intervention

	Mean number of requirement errors ±SD (n)			
	Baseline	During intervention	Reduction in Errors	*p* value
All charts	4.6 ± 1.3 (420)	3.4 ± 1.7 (159)	−1.2 (95%CI 0.9, 1.5)	< 0.001
Weekday charts	4.5 ± 1.3 (323)	3.3 ± 1.7 (132)	− 1.2 (95%CI 0.9, 1.5)	< 0.001
Weekend charts	4.7 ± 1.4 (97)	4.1 ± 1.5 (27)	−0.6 (95%CI − 0.01, 1.2)	0.09
Primary charts	4.6 ± 1.3 (333)	3.7 ± 1.5 (127)	−0.9 (95%CI 0.6, 1.2)	< 0.001
Re-charts	4.4 ± 1.4 (87)	2.2 ± 1.7 (32)	− 2.2 (95%CI 1.6, 2.8)	< 0.001

**Fig. 1:**
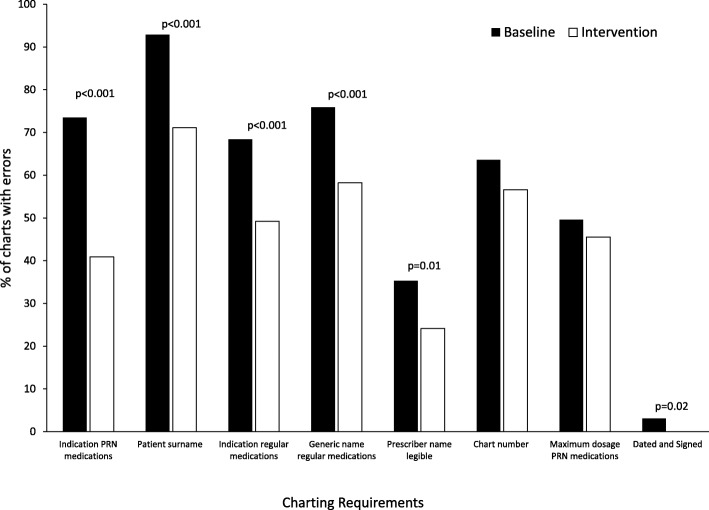
Proportion of charts with errors for each of the eight charting requirements at baseline and during the intervention. The proportion of medication charts (y axis) containing an error for each of the eight charting requirements which are depicted along the x axis. Chi-square examined proportional changes between baseline (closed bars) and the intervention period (open bars). *P* values are reported above the bars

Of the eight charting requirements, the requirement with the highest compliance at baseline was ‘Dating and Signing’ of all prescriptions in the medication charts with error rate of 3.1% of all charts (Table 3 and Fig. 1). The requirement with the lowest compliance at baseline was writing the ‘Patient’s Surname’ below the patient label on the medication chart with an error rate of 92.9% of all charts. The charting requirements with the lowest compliance at baseline were also the requirements which had the most significant reduction during the intervention; ‘Indication for PRN medications’ (32.6% improvement); ‘Patient Surname’ (21.8% improvement); ‘Indication for some regular medications’ (19.2% improvement); ‘Generic name for regular medications’ (17.7% improvement) (Table 3).

**Table 3:** Breakdown of charting requirement errors by error type, from most improved to least improved

	Number of charts with charting requirement errors (%)		
	Baseline *N* = 420	During intervention *N* = 159	*p* value
Indication given for PRN medications	247 (73.5)	45 (40.9)	< 0.001
Patient surname written below label^b^	390 (92.9)	113 (71.1)	< 0.001
Indication given for some regular medications^b^	242 (68.4)	60 (49.2)	< 0.001
Generic name given for regular medications^a^	315 (75.9)	92 (58.2)	< 0.001
Prescriber name printed and legible	148 (35.3)	38 (24.1)	0.01
Chart number given	267 (63.6)	90 (56.6)	0.12
Maximum dosage given for PRN medications	167 (49.6)	50 (45.5)	0.46
Dated and Signed	13 (3.1)	0 (0.0)	0.02

^a^Exceptions were: Movicol, Oxycontin, Endone, Fleet Enema ^b^antibiotics, anticoagulants and steroids including eye drops and topical creams

## Discussion

This pilot study detected a 58% error rate per medication chart for the 8 targeted charting requirements. The multi- interventions approach, including MOWER, visual reminders and training of junior doctors, resulted in a 26% reduction during the intervention. The greatest error rate reduction of 50% was seen in the midweek re-charts. Most improvement was made for those charting requirements that were initially poorly attended to.

We are unaware of any other studies that have used a re-charting approach. However, there are a number of studies that have used a multi-intervention study involving education alongside other interventions such as changes in protocols or feedback to trainees. On the whole, our reduction in error rate of 26% on all charts during the intervention is comparable to these multi-intervention studies [24]. Interestingly, the greatest error rate reduction was seen in the re-charts during the intervention, more than twice the reduction in error rate seen in primary charts, and above the reduction rate found in other studies. There are several possible reasons for these results, one being that it provided another opportunity for the junior doctor to be trained in charting requirements. This agrees with other research that has found that education in combination with audit and feedback is an effective intervention method [25–27]. It could be viewed that in creating the re-charts with a senior and junior doctor team the junior doctor is auditing the primary chart and being given feedback from the senior doctor. A second reason for the improvement could be that by having a team of two looking at the chart this increased accountability and vigilance, which has also been observed in other studies studying team effect [28, 29].

During the intervention our study found that weekend charting practice did not improve, possibly because weekend doctors often cover more than one ward and the doctors completing charts in the medical ward on the weekends may not have been trained in or were made aware of the intervention. As far as we know this is the first study that has compared weekend and weekday charting. The results may indicate the difference in care that is provided on weekends as recognized in higher mortality rates of those admitted to hospitals on weekends [30, 31].

In regards to specific charting errors, we found that the most common type of errors occurring at least once on any given chart were: not providing indication for medications (73% of charts), not providing generic names (76% of charts) and a non-existent or illegible prescriber name (35% of charts) whereas dating and signing was correct in almost all cases. These rates are very similar to other Australian studies [12, 32] but maybe higher than rates in the UK where the majority of errors have been to do with the omission of medications at admission [9, 22, 26]. Lack of indication, using brand names and an inability to identify the prescriber are all errors that can lead to signficant problems particularly when rotations change and new medical personnel are using the charts and are provided with inadequate information. The multi-intervention reduced all of these errors ranging from 15% (prescriber name) to 33% (indications for as needed medications) reductions.

A strength of our study is that the multi-interventions complemented one another and were delivered in different modes (i.e. visual reminders, training, charting practice), so training was reinforced at different times. Multi-interventions with education as a component have been shown previously to be more effective than visual reminders or audit and feedback alone [24, 32, 33]. To facilitate effective implementation of the intervention on the ward, it was vital to have the support of all clinicians regarding the intervention and the overall aims. Key to our success with this was in maintaining a collective focus on enhancing charting practices overall on the ward, rather than a punitive approach of identifying individual prescribers and highlighting their errors. This approach however meant that we were not able to control for individual clinician effects in our analyses. In regards to limitations, although we were able to investigate a number of potential error types we were unable to assess other categories of clinical importance such as listing of adverse drug reactions and route of administration due to a lack of trained staff to interpret these aspects of the chart. However, we feel that a range of criteria was used that reflects overall charting error and the influence of multi-intervention, including both specific medication information such as indication, generic name and maximum dose, and administration information such as chart number and writing the name of the patient, all of which have potentially hazardous outcomes if not followed correctly. We also recognize that the recent introduction of electronic prescribing in this hospital, which occurred after this study was completed, may improve some of the charting error rate. However, it has been found that electronic prescribing is still prone to human error [34–36], which therefore does not preclude the need for a primary care team including a registrar to regularly review inpatient charts. In addition, we were unable to explore prescriber demographics and level of experience in this study, along with patient characteristics. This could be informative for a further study but may affect whether prescribers would be willing to be involved in the intervention.

In regards to further testing and implementing this intervention on a wider scale, for example, in a larger hospital setting, there needs to be involvement and commitment by hospital management as the intervention requires time for staff training on charting practices as well as dedicated time once a week for senior and junior staff to re-chart. In addition, this process would have to be repeated regularly due to staff turnover. Once this is embedded into the organization as a standard practice though, it should happen more naturally, and the net benefit of this time investment would be improved charting and hence better patient care. We acknowledge that there are barriers to changes in practice as junior medical officers are time-pressured in any given health care setting, hence our intervention engaged their immediate seniors rather than relying on pharmacy staff to enforce change. Regardless of location, this is a tool to potentially better ensure each patient’s primary junior medical officer better understands their care by taking full responsibility for their patient’s medications.

## Conclusion

In conclusion, medication chart errors continue to occur on an unacceptable scale. We have shown that a multi-intervention including education of junior doctors, visual reminders and midweek re-charting are effective in improving charting compliance and reducing the rate of charting errors. We advise that a larger study is now conducted using the same multi-intervention MOWER strategy in different ward settings to evaluate feasibility and sustainability of this intervention.

## Additional file

Additional file 1: NIMC Targeted Error Tool. This file depicts the NIMC targeted error tool that was utilized as an educational tool and reminder tool on the wards as described in the methods. (PDF 372 kb)

## Data Availability

The datasets used and analysed during this study are available from the corresponding author on reasonable request.

## Abbreviations

MOWER: Middle Of the Week Everyone gets a Re-chart
NIMC: National Inpatient Medication Chart
