# Supplementary material for: The MOWER (middle of the week everyone gets a re-chart) pilot study: reducing in-hospital charting error with a multi-intervention
Source: BMC Health Serv Res. 2019 Jun 20;19:397. doi: 10.1186/s12913-019-4230-y (PMC6585035; doi:10.1186/s12913-019-4230-y)
Supplement: Supplementary file 1 — NIMC Targeted Error Tool. This file depicts the NIMC targeted error tool that was utilized as an educational tool and reminder tool on the wards as described in the methods. (PDF 372 kb) [file 12913_2019_4230_MOESM1_ESM.pdf]

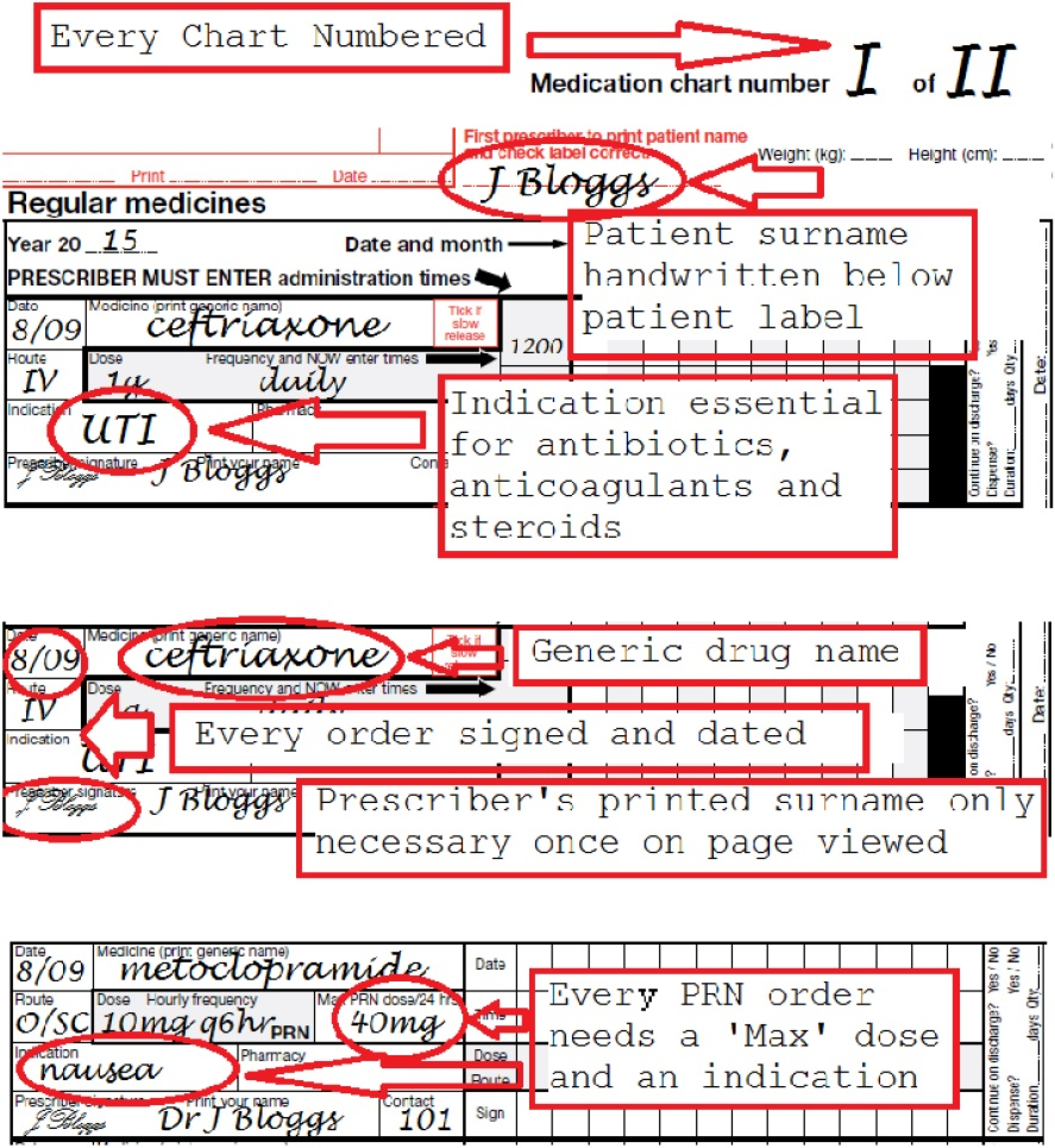
Every Chart Numbered
Medication chart number I of II
First prescriber to print patient name and check label correct
J Bloggs
Weight (kg):
Height (cm):
Print
Date
Regular medicines
Year 20 15
Date and month
PRESCRIBER MUST ENTER administration times
Patient surname handwritten below patient label
Date 8/09
Medicine (print generic name) ceftriaxone
Tick if slow release
1200
Route IV
Dose 1g
Frequency and NOW enter times daily
Indication UTI
Prescriber signature
Print your name J Bloggs
Indication essential for antibiotics, anticoagulants and steroids
Generic drug name
Every order signed and dated
Prescriber's printed surname only necessary once on page viewed
Date 8/09
Medicine (print generic name) metoclopramide
Route O/SC
Dose Hourly frequency 10mg q6hr PRN
Max PRN dose/24 hrs 40mg
Indication nausea
Pharmacy
Prescriber signature
Print your name Dr J Bloggs
Contact 101
Date
Time
Dose
Route
Sign
Every PRN order needs a 'Max' dose and an indication
Continue on discharge? Yes / No
Dispense? days Qty
Duration
